# Psoriasiform Lupus Vulgaris: A Diagnostic Challenge

**DOI:** 10.7759/cureus.69722

**Published:** 2024-09-19

**Authors:** Achala Liyanage, Manuji Bandara, Thusharie Liyanage

**Affiliations:** 1 Faculty of Medicine, University of Ruhuna, Galle, LKA

**Keywords:** cutaneous tuberculosis, extrapulmonary tuberculosis, lupus vulgaris, plaque psoriasis, psoriasiform

## Abstract

Cutaneous tuberculosis is a rare and often overlooked manifestation of tuberculosis, frequently leading to delays from onset to diagnosis. This case report describes the presentation of a 65-year-old male with an asymptomatic solitary plaque with silvery scales persisting for over five years, mimicking solitary plaque psoriasis. Despite intermittent treatment with a potent steroid and a combination of calcipotriol, the patient had only modest alleviation. A skin biopsy was performed, and the histology revealed caseating tuberculoid granulomas. Following treatment with an antituberculosis regimen, the lesion showed significant improvement. The case report highlights the diagnostic challenges of this uncommon entity and the importance of thorough evaluation of recurrent and persistent lesions, particularly in populations with a higher prevalence of tuberculosis. Further, it aims to provide valuable insight to healthcare providers on the atypical clinical presentations of lupus vulgaris mimicking common dermatoses.

## Introduction

Cutaneous tuberculosis (CTB), a rare extrapulmonary variant of tuberculosis, accounts for only 1%-1.5% of all extrapulmonary tuberculosis (TB) infections globally [1-6]. Among CTB forms, lupus vulgaris (LV) is the most common, accounting for approximately 55% of all cases [7]. LV is a chronic, progressive, paucibacillary form of CTB, resulting in a low yield of positive cultures and consequently delayed diagnosis [5,7]. This condition predominantly affects young adults, with a significant prevalence of 41%-68% in children and adolescents and shows a slight female predominance [1, 3, 8]. In Western countries, LV lesions are typically found on the head and neck, whereas in tropical and subtropical regions, they are commonly found on the lower extremities and buttocks [3,8]. This case report describes an elderly male with LV presenting in an atypical site with unusual morphology. It explains the diverse clinical presentations and the diagnostic challenges associated with LV, emphasizing the need for early suspicion of such lesions to prevent long-lasting complications. Additionally, this report contributes to the existing literature by documenting the atypical presentations of LV.

## Case presentation

**Figure 1:**
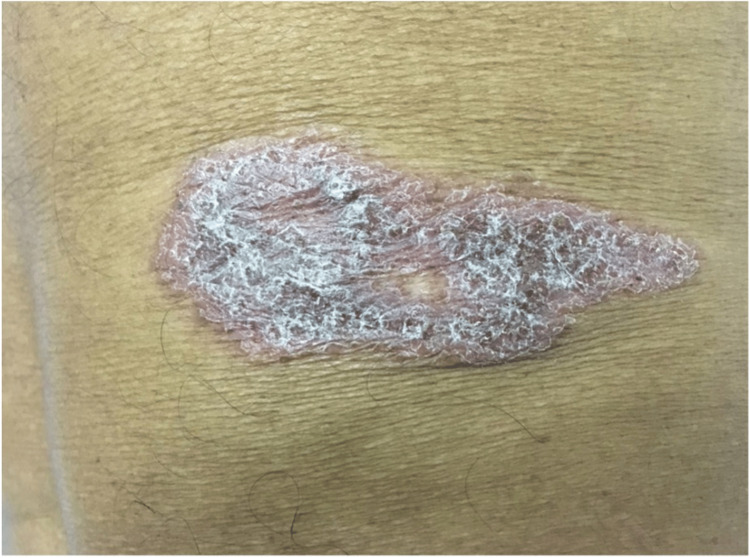
A single well-defined erythematous plaque approximately 7 x 3 cm in size with sharp raised edges and silvery scaling on the surface over the right loin

A 65-year-old male farmer from rural Sri Lanka presented with a single asymptomatic lesion on his right loin that had been recurring for over five years, as depicted in Figure 1. Throughout this period, the lesion partially improved in terms of the degree of thickness and scaling but never completely regressed. Over the years, he had received intermittent treatment with local steroids from private general practitioners, including clobetasol and a combination of calcipotriol and betamethasone for what was initially presumed to be solitary plaque psoriasis. Despite these treatments, the lesion progressively worsened, with periods of remission followed by recurrences at the same site over time. 

The patient did not report any systemic symptoms such as prolonged cough, fever, night sweats, or other constitutional symptoms. He had no prior comorbidities, contact history of tuberculosis, or history of previous trauma.

The physical examination revealed a well-defined, scaly, erythematous annular plaque with silvery scales, measuring 7x3 cm in size. Notably, there were no back dots on the lesion suggestive of spectacle sign as seen in chromoblastomycosis (a differential diagnosis), nail pitting, or onycholysis. Other than a Bacillus Calmette-Guerin (BCG) scar, which was present following vaccination during infancy, his general and systemic conditions, including pulmonary examinations, were normal. He also had no local or distant lymphadenopathy. The Mantoux test was positive showing 10 mm of induration, while the patient’s basic blood count, erythrocyte sedimentation rate, and liver and renal function tests were within the normal range. Tuberculosis-polymerase chain reaction (TB-PCR) testing was not performed due to finite resources.

**Figure 2:**
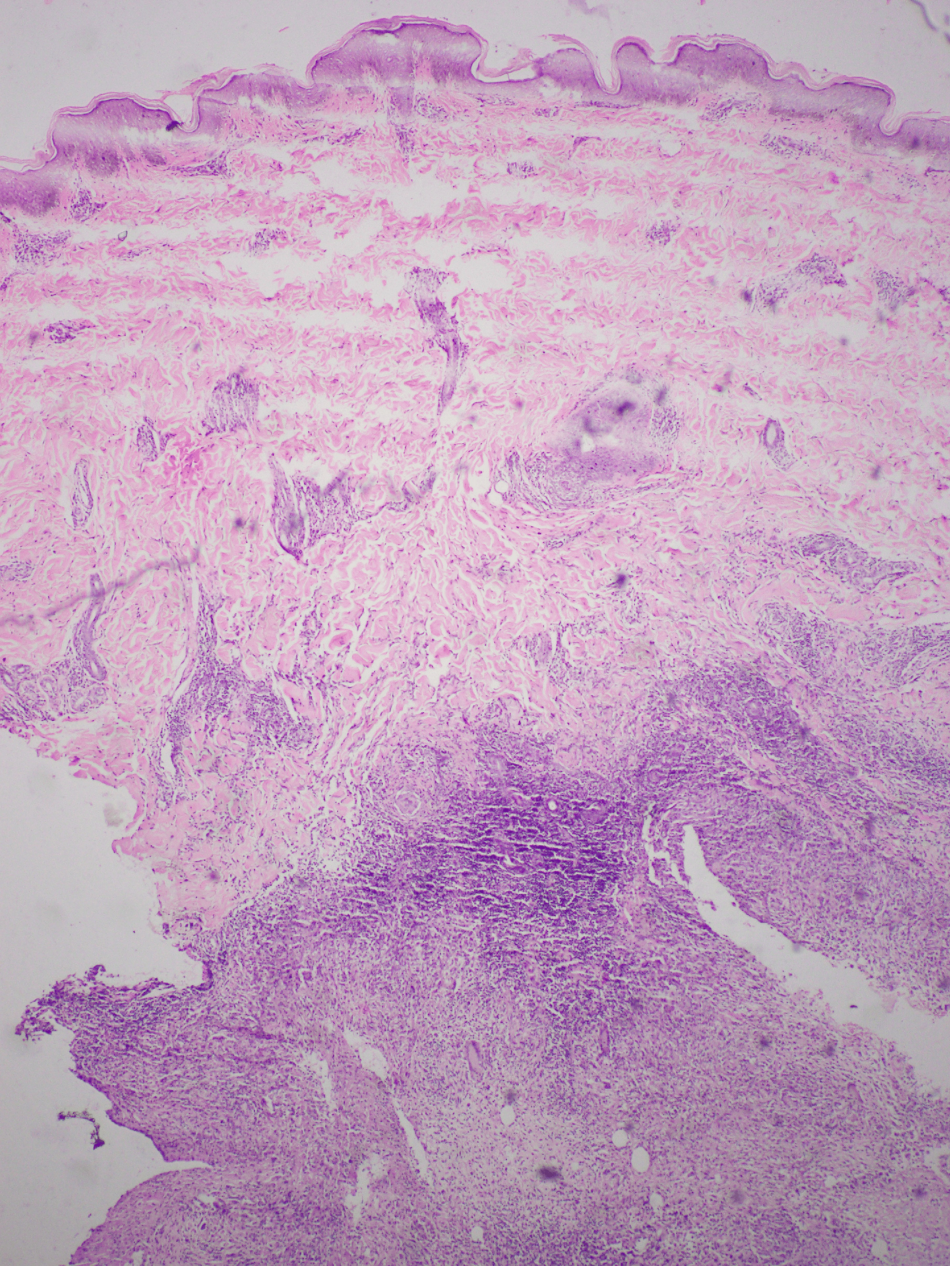
Biopsy showing well-formed granulomas in the mid dermis, surrounded by moderately dense lymphohistiocytic infiltrate with a central necrosis

**Figure 3:**
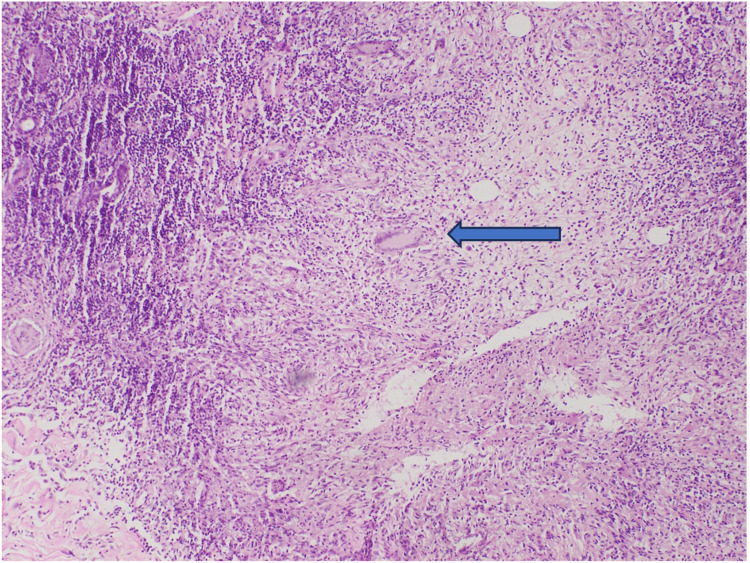
Biopsy showing Langhans giant cells (blue arrow)

Well-formed granulomas with Langhans giant cells and epithelioid cells encircled by caseous necrosis and chronic inflammatory cells were seen in the lesion upon biopsy (Figures 2, 3).

**Figure 4:**
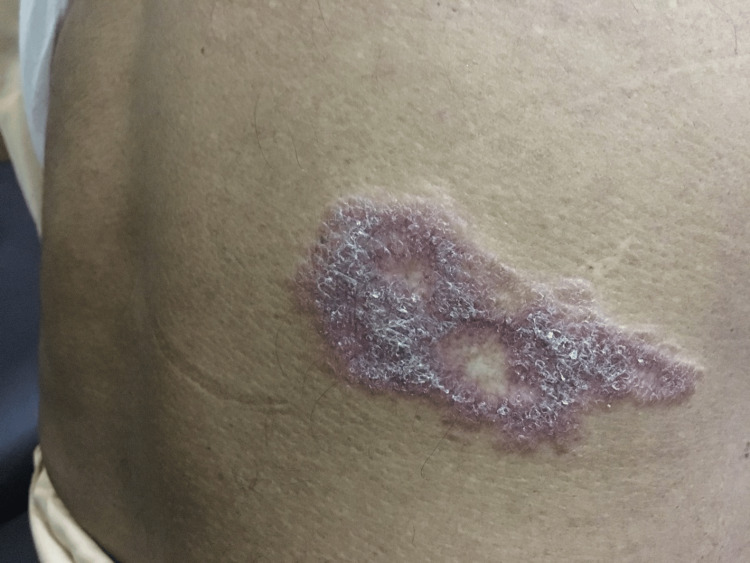
Two months post-treatment: the lesion with reduced silvery scaling and central clearing.

**Figure 5:**
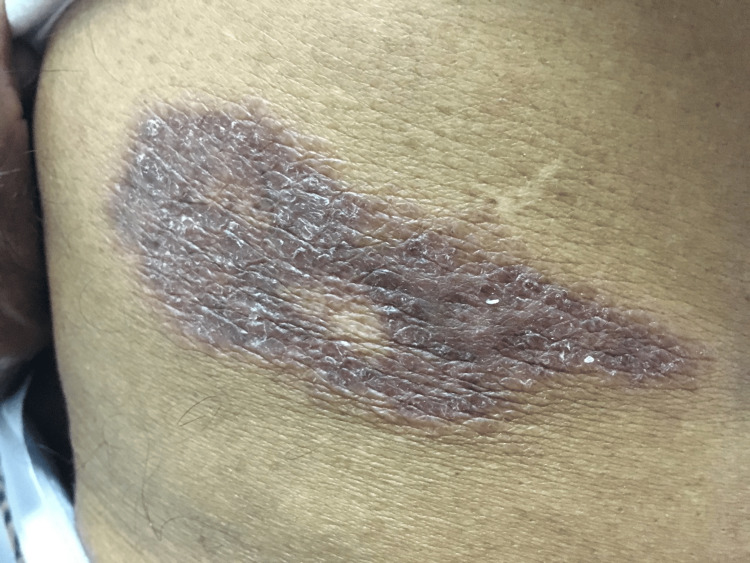
Four months following treatment, the lesion with reduced inflammation and scales with a flattened surface.

**Figure 6:**
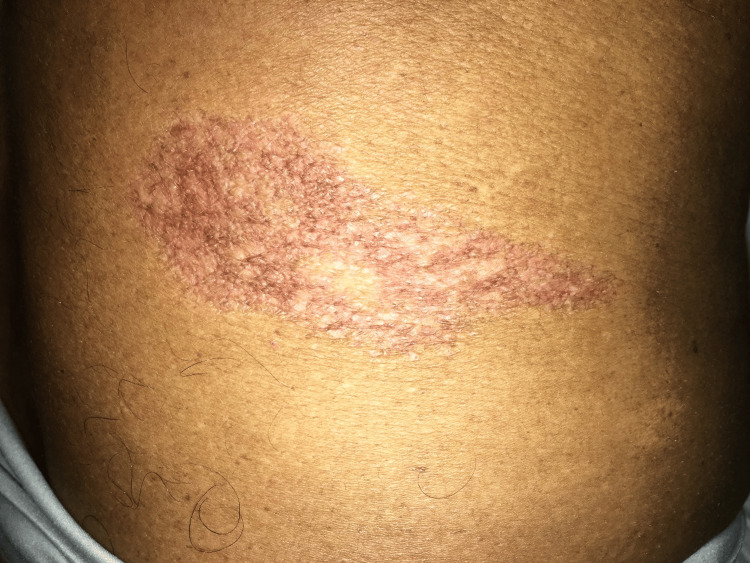
Six months post-treatment: resolution of the lesion with atrophic scarring

Based on these findings, the patient was initiated on an antituberculosis regimen as per the World Health Organization (WHO) recommendations [1-3]. Follow-up assessments were conducted two, four, and six months after treatment to evaluate the progress of the skin symptoms. Serial images of the lesion during treatment (Figures 4, 5) demonstrated significant improvement, with reduced induration and scaling. The margins of the lesion became less defined and inflamed. By six months, the lesion had completely resolved with atrophic scarring, as shown in Figure 6. 

## Discussion

CTB was first documented by Theophile Laennec in 1826 as “prosector’s wart,” an early case of TB verrucosa cutis [3, 8] before Robert Koch isolated the *Mycobacterium tuberculosis* bacillus in 1882 [2]. Despite advances in medical science, CTB remains a significant threat in regions with high tuberculosis prevalence, HIV infections, and multidrug resistance [2]. LV is the most common form of CTB, usually arising from skin reinfection. through endogenous hematogenous or lymphatic spread from an underlying infected focus or exogenous inoculation [9]. The clinical manifestations of LV vary based on the infection route, the patient’s immune status, and prior sensitization to tuberculosis [6]. In the presented case, we were unable to identify a focus of tuberculosis in our patient; however, considering the patient's occupation as an outdoor worker in Sri Lanka, where working shirtless is common, exogenous inoculation remains a plausible cause. 

There are five common clinical manifestations of LV, with the plaque type being the most common, affecting about 32% of all cases [1,3,7]. It begins as discrete red-brown solitary papules that subsequently coalesce into a light-skin-colored plaque [3,9]. Severe forms, such as the ulcerative and mutilating variety, are the most damaging, often leading to severe scarring and deformities, leaving behind an atrophic, crust-like scar. The vegetative form features necrosis and ulceration with less scarring, while other types include the tumor-like, which present as soft-grouped nodules, and the papular nodular type with multiple disseminated lesions [1,3,7]. Rare variants include frambesiform, gangrenous, ulcero-vegetative, lichen simplex chronicus, and sporotrichoid types [1,9]. Multifocal LV lesions are less common but have been reported, especially in patients with multifocal tuberculosis or occasionally in immunocompetent individuals with multiple non-healing plaques at various sites [10,11].

LV often exhibits a gelatinous consistency, described as “apple jelly" on diascopy [1,4,5], which can be difficult to define in individuals with darker skin tones [4,8]. Recent advancements in dermoscopy have improved in detection, showing well-defined telangiectasias on a diffuse yellow-to-orange background with whitish reticular streaks [12]. Histopathological examination remains the gold standard for diagnosis, particularly in endemic regions. Characteristic findings include tuberculoid granulomas, with caseation observed in approximately half of the cases [1, 3]. Pseudoepitheliomatous hyperplasia may also be present [12]. However, the paucibacillary nature of LV complicates diagnosis, often leading to negative cultures. Thus, diagnosis typically relies on Mantoux testing, histopathological appearance, and response to treatment [5,12].

Sarcoidosis, leprosy, warty TB, blastomycosis, chromoblastomycosis, leishmaniasis, lichen planus, psoriasis, lupus erythematous, lymphocytoma, and Bowen’s disease are among the many disorders included in the differential diagnosis for LV [13]. Several reports have documented atypical presentations of LV, including cases misdiagnosed as eczema for 50 years [14], and as a birthmark for 87 years [15]. Furthermore, LV has also been reported in unusual locations, such as the face, mimicking conditions like periorbital cellulitis and basal cell carcinoma [9].

Delayed diagnosis can lead to severe complications, including skin malignancies including (squamous cell carcinoma with a prevalence ranging from 0.5% to 10.5%, and basal cell carcinoma), contractures, scarring, and lymphedema [4,5,9]. Untreated lesions can expand significantly, causing extensive ulceration, tissue destruction, and severe disfigurement [4]. Therefore, early histopathological evaluation is crucial for accurate diagnosis and timely treatment to prevent such complications.

The World Health Organization (WHO) recommends the standard regimen of antituberculosis medication for treating CTB [2,3]. The regimen entails a two-month intensive phase with rifampicin, isoniazid, pyrazinamide, and ethambutol, followed by a maintenance phase lasting four months with rifampicin and isoniazid. Surgical intervention may be required in cases with refractory lesions, significant deformities, or recalcitrant cases [1-3].

## Conclusions

In conclusion, LV continues to pose a significant diagnostic challenge due to its rarity, diverse clinical presentation, paucibacillary natures, and chronicity. This case report highlights how important it is to include CTB in the differential diagnosis of chronic, treatment-resistant lesions, especially in TB-endemic areas. Healthcare providers should maintain a high index of suspicion of CTB not only in areas with high TB prevalence but also in regions with lower prevalence. Recognizing its diverse clinical manifestations is essential for achieving accurate diagnosis and timely treatment.
